# Computational optics for high-throughput imaging of neural activity

**DOI:** 10.1117/1.NPh.9.4.041408

**Published:** 2022-05-20

**Authors:** Yi Xue

**Affiliations:** University of California, Davis, Department of Biomedical Engineering, Davis, California, United States

**Keywords:** computational optics, computer-generated holography, compressive sensing, non-negative matrix factorization, deep learning, neural circuits

## Abstract

Optical microscopy offers a noninvasive way to image neural activity in the mouse brain. To simultaneously record neural activity across a large population of neurons, optical systems that have high spatiotemporal resolution and can access a large volume are necessary. The throughput of a system, that is, the number of resolvable spots acquired by the system at a given time, is usually limited by optical hardware. To overcome this limitation, computation optics that designs optical hardware and computer software jointly becomes a new approach that achieves micronscale resolution, millimeter-scale field-of-view, and hundreds of hertz imaging speed at the same time. This review article summarizes recent advances in computational optics for high-throughput imaging of neural activity, highlighting technologies for three-dimensional parallelized excitation and detection. Computational optics can substantially accelerate the study of neural circuits with previously unattainable precision and speed.

## 1 Introduction

Nearly all aspects of cognition and behavior require the coordinated action of ensembles of neurons that are spread out in a three-dimensional (3D) volume in the mouse brain.^1^^,^^2^ To understand how these ensembles of neurons generate an emergent functional state, we need optical systems that can image these neurons simultaneously rather than sequentially. These optical systems should achieve not only a large field-of-view (FOV) that covers entire ensembles (hundreds of microns to a few millimeters) but also high resolution that can resolve an individual neuron (a few microns). In other words, these optical systems can access large numbers of voxels (hundreds of millions) at a given time, which are considered to be high-throughput systems. The throughput of an optical system is fundamentally limited by the spatial-bandwidth product or the étendue of the system,^3^ which is determined by the degrees-of-freedom (DOF) of the optical hardware. Several optical components could become the DOF bottleneck, such as objective lenses (numerical aperture and magnification), cameras (number of pixels), scanning mirrors (mirror size and maximum scanning angle), and digital light modulators (number of pixels and bit depth). To image the ensembles of neurons in a millimeter-scale volume at micron-scale resolution, optical systems are required to reach hundreds of millions of DOF, whereas current optical systems only have a few millions of DOF.

To overcome the limit of DOF, computational optics that designs optical hardware and computer software jointly becomes a potential choice. The basic idea is to directly measure the significant data while “ignoring” other data. In other words, instead of collecting a huge amount of raw data and processing it afterwards, computational optics extracts the important information during the measurement. To improve imaging speed and signal-to-noise ratio, computational optics sculpts the excitation light to only illuminate the regions-of-interest simultaneously (ROIs, with or without preselection) rather than illuminating the entire volume (Sec. 2). With prior knowledge, computational optics can decompose mixed signals from fewer measurements than conventional methods by solving an inverse problem, which further improves imaging throughput (Sec. 3). In the following review, we introduce how these techniques achieve high-throughput imaging of neural activity *in vivo* and discuss challenges and opportunities in the future.

## 2 Light Sculpture for Parallel Excitation

To study the correlation of neural activity and understand the underlying neural circuits, it is critical to record the activity of multiple neurons simultaneously rather than a single cell at a time. Widefield microscopy that illuminates the entire FOV and captures an image by a camera is the most basic technique for realizing this goal. However, even though widefield microscopy is easy to implement, it does not have z-sectioning ability and stimulates not only fluorescence labeled neurons but also unlabeled areas. These drawbacks increase background noise, reduce the SNR of images, and may cause additional photobleaching and thermal damage. Therefore, selective illumination that only illuminates fluorescence labeled neurons but not unlabeled areas has been developed for imaging multiple neurons simultaneously. Selective illumination can be implemented with either one-photon microscopy^4^^–^^8^ or multiphoton microscopy.^9^^–^^17^ Multiphoton microscopy is more robust to tissue scattering than one-photon microscopy because the excitation light of multiphoton microscopy has a longer wavelength. Therefore, selective illumination generated by multiphoton microscopy has higher precision and less cross-talk when imaging at a few hundred microns deep in the mouse brain. On the other hand, multiphoton microscopy requires higher laser power than one-photon microscopy per excitation spot, so one-photon microscopy can generate two to three orders more spots simultaneously than multiphoton microscopy without causing thermal damage.

To selectively illuminate the ROIs, we need techniques to sculpt light into specific 3D patterns that match with the ROIs. Computer-generated holography (CGH)^18^^,^^19^ is one of the most widely used light-sculpture techniques for selective illumination. CGH modulates the phase of light in the Fourier domain to generate the targeted intensity pattern in the real domain. The commonly used device that can modulate the phase of light is a liquid-crystal-on-silicon (LCoS) spatial light modulator (SLM). CGH has been used for neural imaging^9^^–^^16^ via two approaches. The first approach is scanless CGH microscopy.^9^^–^^14^ This strategy takes an image of the whole FOV first to select the ROIs, then generates multiple foci to illuminate the targeted ROIs and records calcium activity from these areas using a camera without scanning. This approach can simultaneously image multiple neurons located either at the same two-dimensional (2D) plane^9^^–^^12^ or at different planes in 3D.^13^^,^^14^ To image 3D distributed objects with a 2D camera without scanning, pupil encoding in the detection path with a cubic phase plate^13^ or a double-helix phase plate^14^ has been developed to elongate the depth-of-field, so “out-of-focus” signals can be detected as well. The second approach^15^^,^^16^ generates multiple foci located at multiple z-planes using CGH (one focus per plane), and then scans these foci simultaneously on each plane. The fluorescence signal at each scanning location is mixed and detected with a photomultiplier tube (PMT). The mixed signal is decomposed by post-processing, which will be reviewed in the next section. Comparing these two approaches, the scanless approach can image one to two orders more neurons in parallel than the scanning approach, whereas the scanning approach is more immune to tissue scattering and can image deeper inside of the brain than the scanless approach.

The core of CGH technology is the algorithm that computes a 2D phase mask in the Fourier domain for the targeted 3D intensity. This is an ill-posed problem because it tries to control high-dimensional space (3D) with low-dimensional representatives (2D). Therefore, for a given 3D intensity pattern, the corresponding 2D phase mask is not unique because there is no unique solution to an ill-posed optimization problem. Therefore, we need to consider several factors in terms of selecting the best algorithm. First, the algorithms have different computational complexity, that is, the time to compute the phase mask of the same targeted intensity pattern is different. Second, the phase masks computed by different algorithms have different diffraction efficiency, which measures how much light is contributed to the diffraction order versus the zero order. Third, the intensity patterns generated by these phase masks have different accuracy, referring to the similarity between the synthesized intensity pattern and the targeted pattern. Optimized phase masks should efficiently diffract light and generate high-contrast intensity patterns that accurately excite targeted neurons while mitigating the cross talk between target neurons and nontarget neurons.

**Fig. 1:**
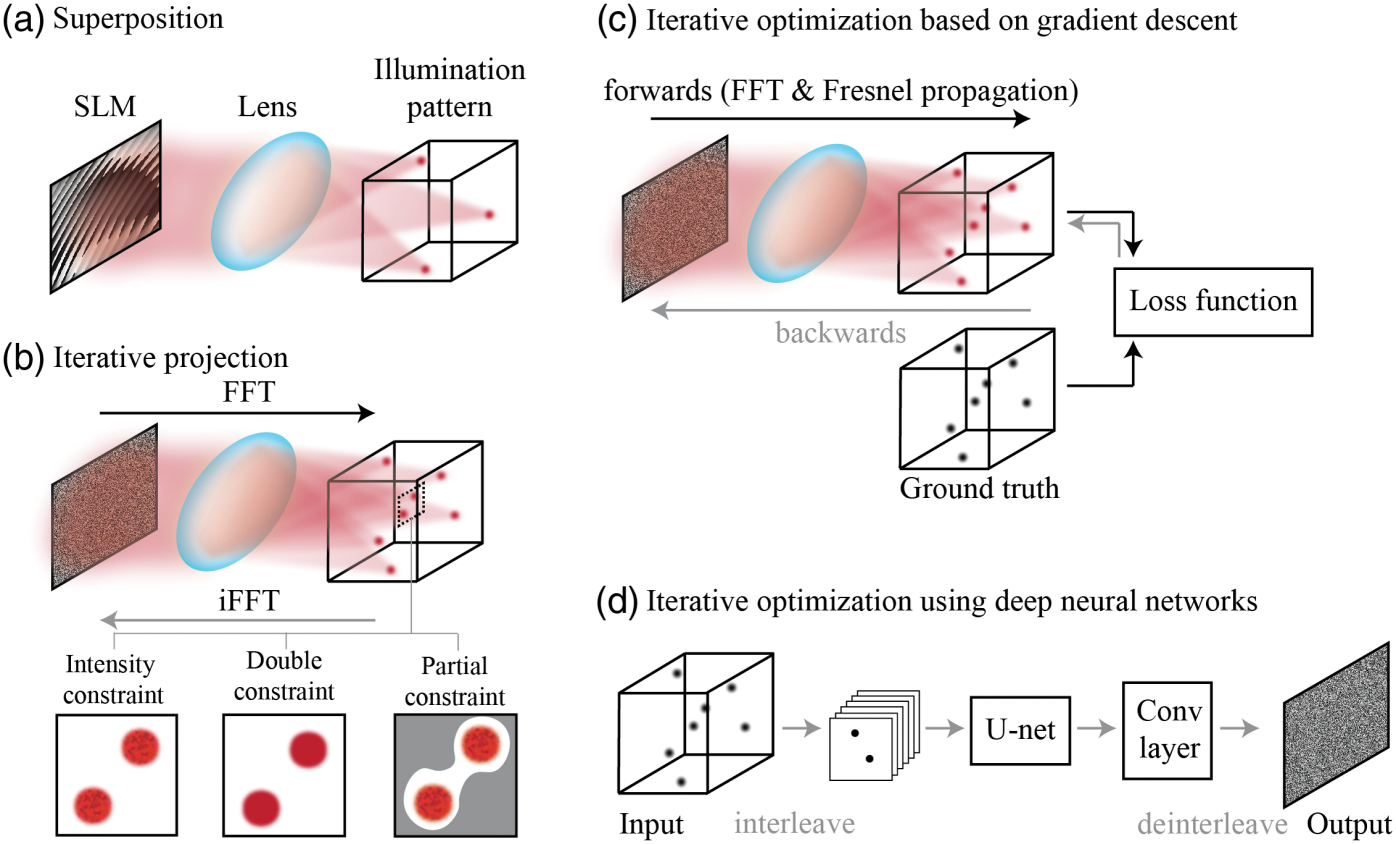
Algorithms for 3D CGH. (a) Superposition algorithms^20^^,^^21^ generate a phase mask for each focus of the illumination pattern without considering interference. (b) Iterative projection algorithms^22^^–^^25^ update the phase mask by iteratively constraining the intensity of illumination patterns in the real domain while leaving the phase of illumination patterns unrestricted. The double-constraint GS algorithm^26^ restricts both the intensity and the phase of illumination pattern to mitigate speckles. Partial constraint algorithms^27^ add error compensation and define unrestricted areas (gray) to improve the performance in the restricted areas (white). (c) Iterative optimization algorithms^28^^,^^29^ build a differentiable forward model and customize the loss function to optimize the phase mask. The optimization problem can be solved by various gradient descent algorithms. (d) The optimization problem of CGH also can be solved by DNNs that generate high accuracy phase masks at fast speeds.^30^^,^^31^

The first category of CGH algorithms is superposition algorithms^20^^,^^21^ [Fig. 1(a)], which split the targeted 3D intensity into multiple 2D planes and compute phase masks for each plane, respectively. The sum of these phase masks becomes the final phase mask for the 3D targeted intensity. Superposition algorithms are highly computationally efficient and good for generating very sparsely distributed foci. However, they have low diffraction efficiency when the number of z-planes increases, given the algorithms treat each axial plane independently without considering the interference between planes.

The second category of CGH algorithms is iterative projection algorithms^22^^–^^25^ [Fig. 1(b)], and the widely used Gerchberg–Saxton (GS) algorithm falls into this category. The GS algorithm iteratively updates the phase mask by propagating the approximate phase mask to the real domain, applying the intensity constraint to it, and then backpropagating the modified complex field to the Fourier domain. The iterative projection algorithms are computationally efficient, but the diffraction efficiency of the approximate phase mask varies a lot depending on the targeted intensity patterns. To generate phase masks that are optimized for the ROIs, partially constrained GS has been developed;^27^ it defines unrestricted areas for flexibility [Fig. 1(b), right in the bottom row]. Also, because CGH generates intensity patterns by coherent interference, speckles are commonly seen in the patterns [grids in the bottom row in Fig. 1(b)]. To remove the speckles, one hardware-based solution is to add a rotating diffuser in the excitation path to make the excitation light partially coherent,^32^ and the software-based solution is to use double-constraint GS that restricts both the intensity and the phase of the targeted pattern [Fig. 1(b), middle in the bottom row].^26^ However, even with these constraints, it is hard to effectively optimize the phase masks for complicated intensity patterns because these algorithms do not explicitly define the loss function.

The third category of CGH algorithms is based on iterative optimization^28^^,^^29^ [Figs. 1(c) and 1(d)]. These algorithms first build a differentiable forward model to formulate the CGH process and customize the loss function, then compute the gradient, and finally solve the phase retrieval problem by gradient descent. Compared with the GS algorithm, the iterative optimization algorithms generate higher quality phase masks that mitigate cross talk and improve the contrast of holography patterns. However, these algorithms based on iterative gradient descent take a long time to converge, so the computational efficiency is lower than the iterative projection algorithms. To improve computing speed, recent works^30^^,^^31^ apply deep neural networks (DNNs) to solve the phase retrieval problem of 3D CGH [Fig. 1(d)]. The approach utilizes an unsupervised U-net to optimize the 2D phase mask for the targeted 3D intensity iteratively. After training, the algorithm can compute the phase mask of an untrained intensity pattern within milliseconds. This approach also can flexibly customize the loss function by adding different penalties to different areas, similar to the partial constraint GS algorithm (“bright areas,” “dark areas,” and “unrestricted areas”). We also can apply a sparsity constraint to the loss function, which improves the contrast of the synthesized intensity pattern.^31^

The performance of CGH can be improved not only by algorithms but also by hardware. For example, CGH can generate diffraction-limited spots for high-resolution imaging. However, when applying CGH to photostimulation, submicron-scale spots may be too small to efficiently draw photocurrent and induce action potentials. Therefore, CGH can be combined with either scanning-based methods^33^^–^^35^ or patterning-based methods^32^^,^^36^^–^^38^ to tailor the focal spots to the shape of neurons. The scanning-based methods place scanning mirrors at the relayed plane of the SLM that scan each diffraction-limited spot generated by CGH across the soma of each neuron.^33^^,^^34^ Compared with a diffraction limited spot, these methods stimulate more opsins and induce larger photocurrent. On the other hand, the patterning-based methods can sculpt light either in the x−y dimension to match the shape of targets, such as the soma or the dendrites of neurons,^36^ or in the z dimension to reduce the out-of-focus light and cross talk.^32^^,^^37^^,^^38^ More details about these two-photon CGH techniques for photostimulation can be found in recent reviews,^39^^,^^40^ and here we focus on the computational part of CGH for imaging.

**Fig. 2:**
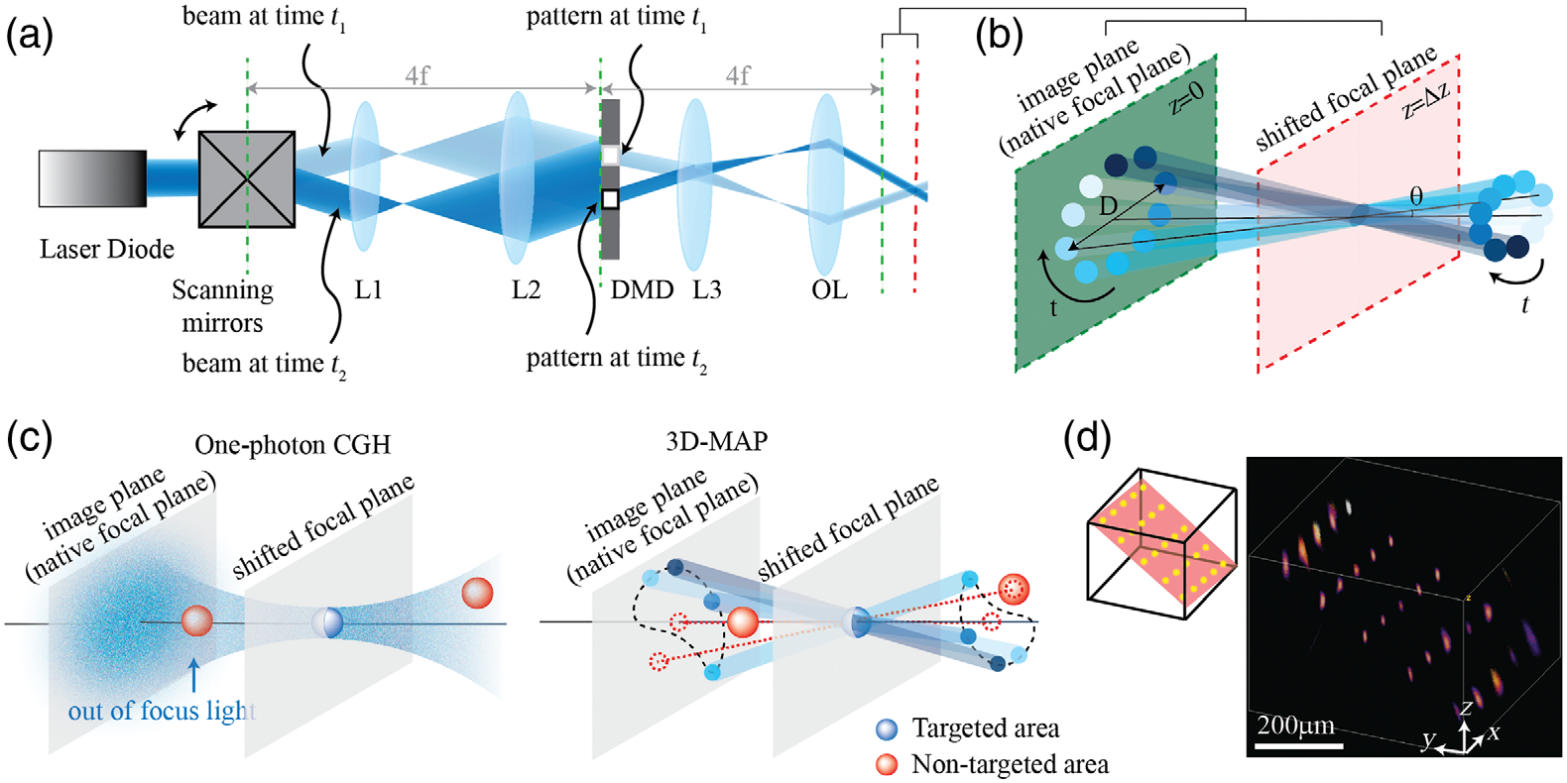
An example of synthesizing 3D intensity patterns by 4D light field control. Images reproduced with permission from 3D multisite random access photostimulation (3D-MAP).^42^ (a) A collimated laser beam illuminates the surface of a DMD with a custom illumination angle set by scanning mirrors. The DMD is synchronized with the scanning mirrors to match the 2D mask of the spatial aperture to the illumination angle. (b) Zoomed in view of the overlapping amplitude masks and illumination angles at the relayed image plane (green) showing how synchronized illumination angles and amplitude masks can generate a focused spot away from the native focal plane (red). Circular patterns labeled by different colors are spatial apertures projected at different times. (c) A focus generated by CGH stimulates the targeted area (blue) in focus but also stimulates non-targeted areas (red) out of focus. 3D-MAP can stimulate only the targeted areas and avoid non-targeted areas by closing the amplitude apertures along propagation directions that project to non-targeted areas (dashed red line). (d) Left: 3D-MAP can simultaneously generate multiple spots in 3D. Right: Experimental measurement of the corresponding 3D fluorescence distribution using a substage camera with a thin fluorescence slide.

However, because CGH is an ill-posed problem, the computed phase mask is not guaranteed to be the best solution. As targeted patterns become more and more complex, such as patterns spanning across many axial planes, the quality of the phase masks drops and the computing time increases. To tackle this problem, four-dimensional (4D) light field modulation has been developed as an alternative to CGH for 3D light sculpture.^41^^,^^42^ Light field modulation controls not only the phase of light $\left(k_{x},k_{y}\right)$ but also the intensity of light (x,y) in the same domain, that is, it modulates light on a 4D hyperplane. Therefore, computing the 4D light field of the targeted 3D intensity is a well-posed problem, so the solution is unique and the computing speed is much faster than for CGH. Early works apply 4D light field control to 3D photography using a digital micromirror device (DMD) combined with a microlens array.^41^ We recently demonstrated 4D light field control for multisite 3D photostimulation *in vivo.*^42^ We first compute the light rays to generate the multiple targets; then we modulate light projection angle with a pair of scanning mirrors and modulate light amplitude with a DMD at the relayed image plane simultaneously [Fig. 2(a)]. To create a focused spot at the relayed image plane [green plane, Figs. 2(a) and 2(b)], we can “open” the corresponding pixels on the DMD; to create a focused spot outside of the native image plane [e.g., red plane, Figs. 2(a) and 2(b)], we “open” a series of apertures on the DMD [blue circles in Fig. 2(b)] sequentially, synchronized with the corresponding projection angle as the scanning mirrors sweep. To generate multiple spots simultaneously, we superpose the patterns corresponding to each target on the DMD. Such 4D modulation has 10 times more DOF than CGH, so it can synthesize patterns that are infeasible for CGH [Fig. 2(c)]. Also, because the DMD can project patterns in tens kilohertz whereas LCoS-SLM can only project patterns in hundreds of hertz, in addition to the computing speed, the patterning speed is also a few orders faster than for CGH. We demonstrated the ability to simultaneously generate 25 foci at custom (x,y,z) locations in a $744\times 744\times 400\;\mu m^{3}$ volume [Fig. 2(d)]. We also use 3D-MAP to interrogate neural circuits in 3D and demonstrate simultaneous photostimulation and imaging of dozens of user-selected neurons in the intact mouse brain *in vivo*. However, the amplitude modulation only allows part of input light to pass through and blocks the residual, so it only has been demonstrated in one-photon microscopy but not yet in multiphoton microscopy.

## 3 Undersampled Detection and Lossless Reconstruction

While computational optics offers customized illumination in the excitation path, it also enables lossless reconstruction from undersampled measurements. This idea is based on the fact that most data that we acquired are compressiable, that is, the data are sparse in some domains. Sparse here refers to the principle of transform sparsity.^43^ If the object of interest $x(x\in R^{m})$ can be represented by an orthonormal basis ($\phi_{i},i=1,2,\ldots ,m$), such as a Fourier basis, the transfer coefficients $\theta_{i}=\langle x,\phi_{i}\rangle$ are sparse when they satisfy

$${\left\|\theta \right\|}_{p}\equiv {\left(\underset{i}{\sum }{\left|\theta_{i}\right|}^{p}\right)}^{1/p}\leq R,$$

where 0<p<2,R>0. Natural signals and images obey the constraints. For example, JPEG is a commonly used image format that usually achieves 10-fold compression without perceptible loss of image quality.^44^ Because the data are compressible, it is not necessary to acquire the data that we will throw away, but rather we should directly measure the data that represent the key information of the object. This strategy allows us to achieve much faster imaging speed over a much larger number of pixels than conventional methods (depending on the compression ratio, it can be over 100:1^44^), overcoming the limit by the Nyquist–Shannon sampling theorem.

**Fig. 3:**
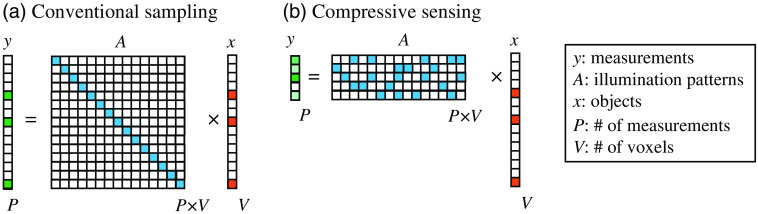
Comparison between conventional sampling and compressive sensing in the spatial domain. (a) Conventional image techniques sample the entire object x, so the number of measurements (P) equals the number of voxels (V). (b) Compressive sensing^45^^–^^47^ encodes the unknown object x with a known illumination pattern (A), and the raw measurement y is a linear combination of multiple voxels of the object. To decompose these voxels of the object, we can solve the inverse problem based on this forward model with the prior of sparsity.

When applying this idea to microscopy, we not only can exploit the natural sparsity of the object but also can control the illumination to further improve the compression ratio. Applying structured illumination to encode the spatial information of imaging object and reconstructing images from undersampled measurements are called compressive sensing^45^^–^^50^ (Fig. 3). Instead of directly imaging the object [Fig. 3(a)], compressive sensing images the inner product of the object and the illumination patterns [Fig. 3(b)]. Multiple random patterns [known, matrix A in Fig. 3(b)] are projected by light sculpture techniques to encode the spatial information of the object, and the emitted light is mixed and recorded by a single-pixel detector [Fig. 3(b), y]. The original structural information [Fig. 3(b), x] can be calculated by solving an inverse problem of this forward model. Therefore, using compressive sensing, the number of voxels in the reconstructed image is no longer limited by the number of pixels on the detector but by the number of independent spatial components. As shown in Fig. 3, the number of measurements P using compressive sensing can be much smaller than that using a one-to-one sampling strategy (5 measurements v.s. 15 measurements, compression ratio is 3). Therefore, compressive sensing is able to improve the imaging speed using fewer measurements and, more importantly, to break the throughput limit set by the number of pixels of detectors.

Given these advantages of compressive sensing, it has been applied to both structural and functional neural imaging. For example, sparsity in spatial and temporal domains is a popular prior used in computational calcium imaging.^51^^–^^54^ Neural activity is typically sparse in the temporal domain, and in the spatial domain, sparsity can be achieved by either the expression of calcium indicators or the structured illumination reviewed in the last section. Instead of detecting calcium activity from a single neuron at a time, computational optics can detect mixed calcium signals from multiple neurons simultaneously and decompose these signals using post-processing algorithms given the prior knowledge of sparsity. Because this approach requires fewer measurements, it can largely improve the imaging speed and system throughput. For example, Pégard et al.^51^ demonstrated simultaneously imaging over 800 neural structures at 100 Hz in a live zebrafish with compressive light-field microscopy.

**Fig. 4:**
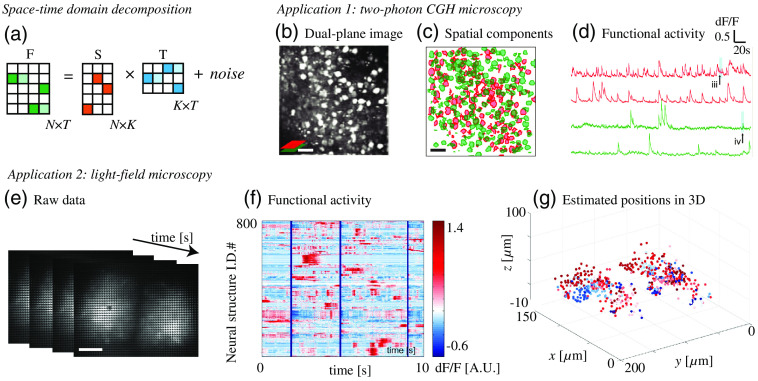
Space–time domain decomposition can be adopted to various optical microscopy systems for functional brain imaging. (a) The key step of space–time domain decomposition algorithms. The mixed measurement F is decomposed into a product of two low-rank matrices containing spatial components (S) and temporal components (T), respectively. (b)–(d) Two-photon CGH microscopy uses constrained NMF to demix the calcium activity of individual neurons from dual-plane overlapping images. Scale bar, 50  μm. (e)–(g) Compressive light-field microscopy with ICA and NMF demonstrates functional imaging of 800+ neural structures at a 100 Hz volumetric sampling rate in a live zebrafish. Scale bar, 50  μm. Images reproduced with permission from: (b) Ref. 15 and (c) Ref. 51.

Another technique leveraging sparsity is space–time domain decomposition for functional imaging. When projecting random illumination patterns sequentially, both spatial and temporal signals from multiple neurons are encoded and mixed in the raw images. To identify the location and calcium activity of each neuron, various matrix decomposition algorithms, such as independent component analysis (ICA)^55^^,^^56^ and nonnegative matrix factorization (NMF),^53^^,^^57^^,^^58^ have been exploited. ICA is a linear demixing method and has been widely used, but it performs worse than NMF when the linear demixing matrix is unavailable such as when neurons spatially overlap. NMF can handle spatially overlapped signals and can decompose the raw measurements (an N×T matrix) into a product of two low-rank matrices (one is N×K and the other one is K×T), containing spatial and temporal information, respectively [Fig. 4(a)]. Here, N is the number of voxels of the image, T is the number of frames, and K is the number of independent components. Note that independent components are not guaranteed to be individual neurons because functional connections between neurons are common. A good initial guess of K is critical to the performance of NMF. When scattering is weak and structural images are available, the initial value of K can be estimated by image segmentation of the structural images; otherwise, the initial value of K can be estimated by greedy algorithms,^15^^,^^53^ ICA,^51^ or singular value decomposition.^58^ In practice, NMF algorithms usually apply constraints as regularizers, such as background constraint or sparsity, to improve the performance.

Space–time domain decomposition algorithms are versatile and can combine with various optical microscopy systems for high-throughput functional imaging. For example, as we mentioned above, two-photon CGH microscopy can scan two axial planes simultaneously and exploit constrained NMF to demix the calcium activity of overlapping neurons [Fig. 4(b)]. NMF also can be combined with other volumetric imaging techniques such as light-field microscopy. Light-field microscopy captures an image of 4D light field by placing a microlens array at the image plane and placing a camera at the focal plane of the microlens array.^59^ Conventional light-field microscopy suffers from tissue scattering, but recent works overcome this challenge by applying space–time domain decomposition algorithms to light-field microscopy^51^^,^^54^^,^^60^ [Fig. 4(c)]. These algorithms treat distortions caused by aberration and scattering as the “signatures” of spatial components, so they can distinguish independent calcium activities through scattering tissue without explicit structural imaging.^51^^,^^58^

## 4 Future Outlook

Computational optics has been exploited for high-throughput, high-speed, and high spatiotemporal resolution neural imaging, but some challenges still hinder the technology from being applied to broader applications.

First, photon starving is always a challenge for neural imaging, and sometimes it is even more severe with computational optics because of the choice of detectors. Parallel and multiplexed detection usually rely on cameras rather than single-pixel detectors, which induce more readout noise and lower SNR at low photon counts. One possible solution is to use multianode PMTs or ultrafast EMCCD cameras that offer multiple pixels and extremely low readout noise at the same time, but the number of pixels is still far less than that of a typical camera. Another possible solution relies on DNNs for image reconstruction at low photon counts.^61^^,^^62^ For example, we have demonstrated that a supervised convolutional neural network can improve the SNR of images taken through scattering tissues,^61^ and a recent work also demonstrates the reconstruction of low-light signals for voltage imaging using a self-supervised denoise algorithm.^62^ In addition, developing lasers that are tailored to neural imaging and developing fluorescence probes with higher quantum efficiency and longer wavelength than current probes will also improve the SNR of images.

Second, the memory limitation of graphics processing units (GPUs) is an emerging challenge as we pursue high-throughput neural imaging. Image data, especially these carrying temporal dynamics, can easily become huge and are infeasible with current algorithms due to the limit of memory. Running out of memory becomes a common error when a GPU tries to handle such big data. Even splitting the big image data into multiple small image files and processing them in parallel, the overall time of image processing could take hours to days. One way to reduce the size of raw data is to only store on-demand information. For example, to record neural activity of multiple neurons, it is probably not necessary to store explicit structural images if we could localize the temporal traces of each independent components from the raw data, which is the idea of NMF we discussed above. The other possibility is to use memory-efficient algorithms that have been developed for physics-based learning.^63^ Also, more powerful computer hardware, such as the tensor processing unit with TensorFlow, can provide more storage and faster computing speed.
